# A 5′-flanking region polymorphism in toll-like receptor 4 is associated with gastric cancer in a Chinese population^☆^

**DOI:** 10.1016/S1674-8301(10)60017-6

**Published:** 2010-03

**Authors:** Hua Huang, Juan Wu, Guangfu Jin, Hanze Zhang, Yanbing Ding, Zhaolai Hua, Yan Zhou, Yan Xue, Yan Lu, Zhibin Hu, Yaochu Xu, Hongbing Shen

**Affiliations:** a Department of Epidemiology and Biostatistics, Cancer Center, Nanjing Medical University, Nanjing 210029, Jiangsu, China; b Department of Gastroenterology, Yangzhou First People's Hospital, Yangzhou 225009, Jiangsu, China; c Department of Epidemiology, Yangzhong Cancer Institute, Yangzhong 212200, Jiangsu, China; d Department of Oncology, Yixing People's Hospital, Yixing 214200, Jiangsu, China

**Keywords:** toll-like receptor 4, polymorphisms, susceptibility, gastric cancer

## Abstract

**Objective:**

Inflammation induced by *H.pylori* colonization in the stomach is related to the development of gastric cancer and the genetic variations of the genes involved in the immune responses modify the host response to the infection. The aim of this study was to evaluate whether polymorphisms in the *toll*-*like receptor 4 (TLR4)* gene, a key regulator of both innate and adaptive immunity, were related to the susceptibility to gastric cancer in a Chinese population.

**Methods:**

Two variations in the 5′-flanking region of *TLR4* (rs1927914 T > C and rs10759932 T > C) were genotyped by using the PCR-restriction fragment length polymorphism (RFLP) assay in a case-control study of 1,053 incident gastric cancer cases and 1,100 cancer-free controls in a Chinese population.

**Results:**

Individuals carrying the C allele of rs10759932 had a significantly reduced risk of gastric cancer (adjusted OR = 0.81; 95%CI = 0.67-0.96), compared with the wild-type homozygote (TT), and the protective effect was not significantly different among subgroups stratified by age, sex, smoking, drinking and *H.pylori* infection status (*P* for heterogeneity > 0.05). No significant association was observed between rs1927914 and gastric cancer risk in this study population.

**Conclusion:**

The T to C allele substitution of rs10759932 may play a protective role in gastric carcinogenesis in a Chinese population. Large studies with different ethnic populations are warranted to confirm these findings.

## INTRODUCTION

*Helicobacter pylori* (*H. pylori*), a gram-negative microaerophilic bacterium, mainly colonizes the human stomach and induces various gastric lesions, ranging from gastritis to cancer. Generally, most individuals with *H. pylori* infection develop chronic gastritis, but only few of them (<1%) ultimately result in gastric cancer. Therefore, it is well recognized that host factors, especially genetic heterogeneity, may be important in gastric carcinogenesis interacting with *H. pylori* infection. Innate immunity plays a crucial role in host protection against pathogens, relying on pattern recognition receptors, such as the toll-like receptors (TLRs), to alter the immune system response to existing or invading bacteria. The TLRs are cell-surface signaling molecules that recognize pathogen-associated molecular patterns expressed on infectious agents. Once activated, the TLRs lead not only to the induction of an inflammatory response, but also to the development of antigen-specific adaptive immunity[1]. *H. pylori* lipopolysaccharide (LPS) is considered to be one of the virulence factors involved in the gastritis, inducing secretion of proinflammatory cytokines, inducible NO and oxygen radicals by activating monocytes and gastric epithelial cells. TLR4, a member of the TLR family and the main receptor of LPS[2],[3], is involved in recognition of *H. pylori* and plays an important role in gastroduodenal diseases and gastric carcinogenesis[3],[4].

Two non-synonymous polymorphisms of *TLR4*, Asp299Gly (rs4986790) and Thr399Ile (rs4986791), have been shown to be extensively involved in *H. pylori* infection-related disease, including gastric cancer. For example, Hold *et al*[5] found that the 399Ile allele was associated with an increased susceptibility to gastric cancer in a multi-stage study, which was supported by some other studies[4],[6],[7], although there were some conflicting results[8]. However, the above two single nucleotide polymorphisms (SNPs) were absent in populations of eastern Asians[9]–[11], including Chinese[10]. Alternatively, we found two common polymorphisms (minor allele frequency≥0.05, rs1927914 and rs10759932) located in the 5′ flanking region of the *TLR4* gene in a Chinese population by searching the NCBI SNP database, and these two polymorphisms may influence transcriptional factor binding site and modify the promoter activity and gene expression. In the present study, we hypothesized that the above two SNPs in *TLR4* were associated with the risk of gastric cancer in the high-risk Chinese population. To test this hypothesis, we performed genotyping analysis for the two SNPs in the 5′-flaking region of *TLR4*, rs1927914 and rs10759932, in a case-control study with 1,053 gastric cancer cases and 1,100 cancer-free controls from a high incidence area of gastric cancer, Jiangsu Province, China.

## MATERIALS AND METHODS

### Study subjects

Incident patients with histologically confirmed gastric adenocarcinoma were consecutively recruited from Jiangsu Province, including the cities of Yangzhong, Yixing, Yangzhou and Nanjing, between January 2003 and August 2007. Gastric cardia cancers were defined as tumors located within 20 mm distal to the gastro-esophageal junction, as we previously described[12]. The pathohistology was classified according to Lauren's classification[13] by senior pathologists. The exclusion criteria included reporting previous cancer history and having undergone radiotherapy or chemotherapy. The cancer-free controls were selected from individuals living in the same residential areas as the cases and were frequency-matched to the cases by age (±5 years) and sex. All the subjects were genetically unrelated Han Chinese.

Each participant was scheduled for an interview after a written informed consent was obtained and a structured questionnaire was administered by trained interviewers to collect information on demographic data and environmental exposure history including tobacco smoking and alcohol consumption. Those who had smoked <1 cigarette per day for less than 1 year in their lifetime were defined as non-smokers, otherwise, they were considered as smokers, and those who consumed 3 or more alcohol drinks a week for over 6 months were considered as alcohol drinkers. After an interview, an approximately 5-ml venous blood sample was collected from each subject. The serum antibodies against *H. pylori* CagA was determined by enzyme-linked immunosorbent assay (rat anti - human Cytotoxin Associated Gene A IgG, Adlitteram Diagnostic Laboratories, Shanghai, China) in 767 cases and 916 controls based on the availability of serum samples of the subjects. The cutoff value for positive and negative results was determined according to the manufacture's instructions. The OD value≥3.5 was defined as serum positive. The study was approved by the Institutional Review Board of Nanjing Medical University.

### SNP selection and genotyping

The NCBI public SNP database was used to search all common (minor allele frequency≥0.05 in Chinese population) and potentially functional polymorphisms of the *TLR4* gene, i.e., located at the 5′ flanking region, 5′ untranslated region or exons with amino acid change. Two SNPs in the 5′ flanking region (rs1927914 T > C and rs10759932 T > C) were finally selected.

The SNP rs1927914 T > C was genotyped by the PCR-restriction fragment length polymorphism (RFLP) method. The primers were sense-5′-TGGGATTAAATGAACTGG-3′ and antisense-5′- TGCTTGGAGGATATTACAG-3′, which generated a 155 bp product. The PCR product was then digested by *Nla* III (Fermentas International Inc, Canada) and separated on a 3% agarose gel stained with ethidium bromide. The C allele results in two fragments of 116 bp and 39 bp, and the T allele produce one fragment of 155 bp. The rs10759932 T > C variation was detected by a primer-introduced restriction analysis (PIRA)-PCR assay and a mismatched C was introduced to the anti-sense primer to replace T at + 2-bp from the polymorphic site to create a *Hha* I restriction site. The primers were sense-5′-TTTGTATAATTTGACTACCATTGCGT-3′ and antisense-5′- CATTTTTTCACATCTTCACCAGC-3′, and the amplified product (139 bp) was then digested by *Hha* I (Fermentas International Inc, Canada). The C allele produced two fragments of 117 bp and 22 bp, while the T allele resulted in a single 139 bp.

Genotyping was performed without knowing the subject's case or control status and the results were independently reviewed by two research assistants. To validate the genotyping results, 10% of the samples were randomly selected to confirm the assays, and the results were 100% concordant.

### Statistical analyses

Goodness-of-fit χ^2^ test was used to test the Hardy-Weinberg equilibrium for each SNP among controls. Differences in selected demographic variables, smoking and drinking status, serum CagA status, and frequencies of the genotypes and alleles between the cases and controls were evaluated by the χ^2^ tests. The association between the genotypes of *TLR4* SNPs and gastric cancer risk were estimated by computing the odds ratios (ORs) and 95% confidence intervals (CIs). The heterogeneity between stratifications was assessed with the Chi-square-based Q test. All the statistical analyses were performed by Statistical Analysis System software (version 9.1.3; SAS Institute, Cary, NC).

## RESULTS

The distribution of selected characteristics between 1,053 gastric cancer patients and 1,100 cancer-free controls are summarized in ***Table 1***. There were no significant differences in terms of distribution in age and sex between the two groups (*P* = 0.45 and 0.21, respectively), suggesting that our frequency-matching was adequate. The mean age for cases was 60.63±10.18 years and 60.05±10.36 years for controls. As shown in ***Table 1***, we did not find significant differences in smoking and drinking status between the cases and controls (*P* = 0.22 for smoking and *P* = 0.29 for drinking). The positive rate of CagA in cases was 28.6% (219/767), while the control group was 25.7% (235/916), and the difference was not statistically significant between the two groups (*P* = 0.18). Among the gastric cancer cases, 472 (44.8%) were cardia gastric cancer, 482 (45.8%) non-cardia gastric cancer, and 99 (9.4%) others (including mixed and unknown). For the histological subtypes, there were 735 (69.8%) intestinal-type, 188 (17.9%) diffuse-type, and 130 (12.3%) others (including mixed and unknown).

The genotype distribution of *TLR4* rs1927914 and rs10759932 in the cases and controls are shown in ***Table 2***. The observed genotype frequencies for the two polymorphisms were both in Hardy-Weinberg equilibrium in the controls (*P* = 0.80 and 0.62, respectively). The rs10759932 genotype frequencies were 56.8% (TT), 34.8% (TC) and 8.5% (CC) in the cases and 51.4% (TT), 41.0% (TC) and 7.6% (CC) in the controls and the difference was statistically significant (χ^2^ = 8.11, *P* = 0.018). The logistic regression analysis revealed that the TC heterozygote was associated with a significantly reduced risk of gastric cancer (adjusted OR = 0.76, 95%CI = 0.63-0.92), compared with the TT wild-type homozygote. When we combined the variant genotypes (TC/CC) assuming a dominant genetic model, TC/CC genotypes were significantly associated with a decreased risk of gastric cancer (adjusted OR = 0.81, and 95%CI = 0.67-0.96). The rs1927914 genotype frequencies were 35.8% (TT), 47.6% (TC) and 16.6% (CC) in the cases, which were very close to those of controls [36.3% (TT), 48.2% (TC) and 15.5% (CC)] (*P* = 0.81). No significant association was detected between rs1927914 genotypes and risk of gastric cancer (adjusted OR = 0.99, 95%CI = 0.82-1.21 for TC vs TT and adjusted OR = 1.09, 95%CI = 0.83-1.43 for CC vs TT, respectively).

In the stratified analysis (***Table 3***), the protective effect of the rs10759932 TC/CC genotype remained significant among young subjects (aged <60), males, drinkers, individuals with the negative serum CagA, and both intestinal and diffuse type cases. However, there were no significant differences between different subgroups stratified by these variables (*P* > 0.05 for heterogeneity test). In addition, we did not find any significant effect for rs1927914 TC/CC genotypes on risk of gastric cancer in different strata (data not shown).

**Table 1.** Comparison of selected characteristics between gastric cancer cases and controls

Characteristics	Cases(*n* = 1053)		Controls(*n* = 1100)		*P*^a^
	n	%	n	%	
Age (years)					
< 60	454	43.1	492	44.7	0.45
≥ 60	599	56.9	608	55.3	
Sex					
Male	748	71.0	754	68.6	0.21
Female	305	29.0	346	31.5	
Smoking status					
No	608	57.7	606	55.1	0.22
Yes	445	42.3	494	44.9	
Drinking status					
No	789	74.9	802	72.9	0.29
Yes	264	25.1	298	27.1	
CagA^b^					
Negative	548	71.4	681	74.3	0.18
Positive	219	28.6	235	25.7	
Tumor site					
Cardia cancer	472	44.8			
Non-cardia cancer	482	45.8			
Other^c^	99	9.4			
Histological types					
Intestinal	735	69.8			
Diffuse	188	17.9			
Other	130	12.3			

^a^ Two-sided χ^2^ test. ^b^ 767 cases and 916 controls were available. ^c^ Other means unknown or mixed type.

**Table 2.** Genotypes distribution of *TLR4* among gastric cancer cases and controls

Genotype	Cases	Controls	OR (95%CI)	Adjusted OR (95%CI)^a^
	N(%)	N(%)		
rs1927914^b^	N = 946	N = 987		
TT	339(35.8)	358(36.3)	1.00	1.00
TC	450(47.6)	476(48.2)	1.00(0.82-1.22)	0.99(0.82-1.21)
CC	157(16.6)	153(15.5)	1.08(0.83-1.42)	1.09(0.83-1.43)
TC/CC	607(64.2)	629(63.7)	1.02(0.85-1.23)	1.02(0.84-1.23)
C allele	764(40.4)	782(39.6)		
rs10759932^c^	N = 909	N = 1053		
TT	516(56.8)	541(51.4)	1.00	1.00
TC	316(34.8)	432(41.0)	0.77(0.64-0.93)	0.76(0.63-0.92)
CC	77(8.5)	80(7.6)	1.01(0.72-1.41)	1.03(0.74-1.45)
TC/CC	393(43.2)	512(48.6)	0.81(0.67-0.96)	0.81(0.67-0.96)
C allele	470(26.0)	592(28.1)		

^a^ Adjusted for age, sex, smoking and drinking status. ^b^ Genotyping failed in 107 cases and 113 controls. ^c^ Genotyping failed in 144 cases and 47 controls.

**Table 3.** Stratified analyses of rs10759932 genotypes between gastric cancer cases and controls

Variables	Cases (*n* = 909)		Controls (*n* = 1053)		Adjusted OR (95%CI)^a^		*P* for heterogeneity
	TT N (%)	TC/CC N (%)	TT N (%)	TC/CC N (%)	TT	TC/CC	
Age (year)							0.23
< 60	231(57.5)	171(42.5)	230(48.8)	241(51.2)	1.00	0.71(0.54-0.93)	
≥60	285(56.2)	222(43.8)	311(53.4)	271(46.6)	1.00	0.89(0.70-1.14)	
Sex							0.85
Female	370(57.1)	278(42.9)	375(52.0)	346(48.0)	1.00	0.82(0.66-1.02)	
male	146(55.9)	115(44.1)	166(50.0)	166(50.0)	1.00	0.79(0.57-1.10)	
Smoking status							0.55
No	302(56.6)	232(43.5)	289(49.7)	292(50.3)	1.00	0.77(0.61-0.98)	
Yes	214(57.1)	161(42.9)	252(53.4)	220(46.6)	1.00	0.86(0.65-1.13)	
Drinking status							0.10
No	389(55.7)	309(44.3)	403(52.6)	363(47.4)	1.00	0.88(0.72-1.08)	
Yes	127(60.2)	84(39.8)	138(48.1)	149(51.9)	1.00	0.62(0.43-0.89)	
CagA							0.07
Negative	267(55.9)	211(44.1)	312(48.6)	330(51.4)	1.00	0.75(0.59-0.95)	
Positive	91(52.9)	81(47.1)	130(56.5)	100(43.5)	1.00	1.15(0.77-1.72)	
Tumor site							0.42
Cardia	221(58.3)	158(41.7)	541(51.4)	512(48.6)	1.00	0.76(0.60-0.96)	
Non-cardia	238(54.8)	196(45.2)	541(51.4)	512(48.6)	1.00	0.87(0.70-1.10)	
Histological types							0.50
Intestinal	369(57.5)	273(42.5)	541(51.4)	512(48.6)	1.00	0.78(0.64-0.96)	
Diffuse	99(60.7)	64(39.3)	541(51.4)	512(48.6)	1.00	0.68(0.48-0.96)	

^a^Adjusted for age, sex, smoking and drinking status.

## DISCUSSION

In this case-control study of gastric cancer, we investigated the associations of two SNPs in the 5′-flanking region of *TLR4* with the risk of gastric cancer in a Chinese population. We found, for the first time, that rs10759932 variant genotypes (TC/CC) of the *TLR4* gene were associated with a significantly reduced risk of gastric cancer in this high-risk population.

TLR4 was identified as a potential binding receptor for *H. pylori* on gastric epithelial cells by recognizing the LPS of the gram-negative bacteria. In the human stomach, *TLR4* is known to be expressed by epithelial cells, which was demonstrated to become greater in *H. pylori* gastritis than in non-inflamed gastric mucosa[14]. TLR4, in conjunction with CD14 and MD-2, transductes signals through MyD88, Toll/IL-1 receptor domain and TRAF6. This promotes transcription of genes, which are involved in immune activation, including the transcription factor NF-kB and MAP kinase pathways[15] The dysregulation of TLR signaling may result in an unbalanced ratio between pro- and anti-inflammatory cytokines and thus induce the development of precancerous lesions and cancer[6],[16] *TLR4* Asp299Gly and Thr399Ile polymorphisms, located in the coding sequence and resulting in amino acid exchanges, were shown to affect the TLR4 extracellular domain and reduce the responsiveness to *H. pylori* LPS[17], while Asp299Gly polymorphism was also observed to increase the pro-inflammatory tumor necrosis factor response[18],[19]. Alejandra *et al*[4] reported that these two SNPs could alter mucosal cytokine and chemokine patterns in Mexican gastroduodenal diseases. Both SNPs have been implicated in the process of gastric cancer and its precursors[4]–[7]. However, these two SNPs are absent or rare in the Chinese population[10],[20],[21] and thus may not be an important factor in determining the outcome of the *H. pylori* infected individuals in such areas.

In this study, we identified two SNPs located in the 5′ flanking region of *TLR4* and found that rs10759932 variant genotypes (TC/CC) were associated with a significantly reduced risk of gastric cancer. It has been demonstrated that the low-functioning *TLR4* polymorphisms may result in a reduced inflammatory response associated with a low-damaging infection resulting in persistent infection[22]–[24], whereas *TLR4* mRNA was up-regulated in gastric epithelial cell lines infected with *H. pylori*[25]. Therefore, it is biologically plausible that the 5′ flanking region polymorphisms may influence the binding affinity of the transcriptional factors, regulate the transcriptional level of *TLR4*, involve the process of inflammatory response and host immunity, and finally modulate the development of gastric cancer. However, functional evaluations need to confirm these hypotheses in further studies.

Cytotoxin-associated gene pathogenicity island (cagPAI), a 40-kb cassette of genes that codes a type IV secretory system (T4SS) involved in the injection of the CagA protein into the epithelial cells, is one of the important virulence factors with regard to *H.pylori* infection. Within the cytosol, CagA is phosphorylated by multiple members of the Src family of kinases, considered to play a key role in carcinogenesis, which are involved in cell proliferation and differentiation[26]. Anti-CagA antibody of *H. pylori* was not observed to modify the development of gastric carcinogenesis in this study. Interestingly, in the stratified analyses, the protective effect of rs10759932 TC/CC genotypes seemed prominent in individuals with negative CagA, although there was not a significant difference between subgroups with different CagA status (*P* for heterogeneity = 0.07). This genetic variation may be associated with other virulence factors or the CagA-negative strain may be protective elements interacting with *TLR4* gene.

Several potential limitations in this study need to be addressed. First of all, because the gastric cancer cases were recruited from the hospitals and the controls were selected from the general community, inherent selection bias cannot be completely excluded. However, potential confounding factors might have been minimized by matching age, sex and residential areas, and further controlled in data analysis with adjustment for age, sex, smoking and drinking status. Secondly, the serum status of *H. pylori* CagA was determined by enzyme-linked immunosorbent assay, which may not represent the whole spectrum of *H. pylori* infection, although CagA is considered one of most important virulence factors. Thirdly, the functional significance of rs10759932 was not determined in this study. Further studies with biological assays are needed to clarify the potential mechanism of the SNP modifying gastric cancer development.

In summary, this study revealed that the SNP rs10759932 in the 5′-flanking region of *TLR4* was associated with gastric cancer risk in Chinese population, which provides further important evidence that genetic variants in the *TLR4* gene contribute to gastric cancer susceptibility. However, large studies with different ethnic populations and functional evaluations are warranted to confirm these findings.

## References

[1] Farinha P, Gascoyne RD (2005). Molecular pathogenesis of mucosa-associated lymphoid tissue lymphoma. J Clin Oncol.

[2] Smith MF, Mitchell A, Li G, Ding S, Fitzmaurice AM, Ryan K (2003). Toll-like receptor (TLR) 2 and TLR5, but not TLR4, are required for Helicobacter pylori-induced NF-kappa B activation and chemokine expression by epithelial cells. J Biol Chem.

[3] Ohara T, Morishita T, Suzuki H, Hibi T (2006). Heterozygous Thr135Ala polymorphism at leucine-rich repeat (LRR) in genomic DNA of toll-like receptor 4 in patients with poorly-differentiated gastric adenocarcinomas. Int J Mol Med.

[4] Trejo-de la OA, Torres J, Perez-Rodriguez M, Camorlinga-Ponce M, Luna LF, Abdo-Francis JM (2008). TLR4 single-nucleotide polymorphisms alter mucosal cytokine and chemokine patterns in Mexican patients with Helicobacter pylori-associated gastroduodenal diseases. Clin Immunol.

[5] Hold GL, Rabkin CS, Chow WH, Smith MG, Gammon MD, Risch HA (2007). A functional polymorphism of toll-like receptor 4 gene increases risk of gastric carcinoma and its precursors. Gastroenterology.

[6] Achyut BR, Ghoshal UC, Moorchung N, Mittal B (2007). Association of Toll-like receptor-4 (Asp299Gly and Thr399Ileu) gene polymorphisms with gastritis and precancerous lesions. Hum Immunol.

[7] Santini D, Angeletti S, Ruzzo A, Dicuonzo G, Galluzzo S, Vincenzi B (2008). Toll-like receptor 4 Asp299Gly and Thr399Ile polymorphisms in gastric cancer of intestinal and diffuse histotypes. Clin Exp Immunol.

[8] Garza-Gonzalez E, Bosques-Padilla FJ, Mendoza-Ibarra SI, Flores-Gutierrez JP, Maldonado-Garza HJ, Perez-Perez GI (2007). Assessment of the toll-like receptor 4 Asp299Gly, Thr399Ile and interleukin-8 -251 polymorphisms in the risk for the development of distal gastric cancer. BMC Cancer.

[9] Noguchi E, Nishimura F, Fukai H, Kim J, Ichikawa K, Shibasaki M (2004). An association study of asthma and total serum immunoglobin E levels for Toll-like receptor polymorphisms in a Japanese population. Clin Exp Allergy.

[10] Wu MS, Cheng TY, Shun CT, Lin MT, Chen LC, Lin JT (2006). Functional polymorphisms of CD14 and toll-like receptor 4 in Taiwanese Chinese with Helicobacter pylori-related gastric malignancies. Hepatogastroenterol.

[11] Tahara T, Arisawa T, Shibata T, Hirata I, Nakano H (2007). Absence of common polymorphisms of toll like receptor 4 (TLR4): Asp299Gly, Thr399Ile in patients with gastroduodenal diseases in Japan. J Clin Biochem Nutr.

[12] Wu J, Lu Y, Ding YB, Ke Q, Hu ZB, Yan ZG (2009). Promoter polymorphisms of IL2, IL4, and risk of gastric cancer in a high-risk Chinese population. Mol Carcinog.

[13] Lauren P (1965). The two histological main types of gastric carcinoma: Diffuse and so-called intestinal-type carcinoma. an attempt at a histo-clinical classification. Acta Pathol Microbiol Scand.

[14] Schmausser B, Andrulis M, Endrich S, Lee SK, Josenhans C, Muller-Hermelink HK (2004). Expression and subcellular distribution of toll-like receptors TLR4, TLR5 and TLR9 on the gastric epithelium in Helicobacter pylori infection. Clin Exp Immunol.

[15] Takeda K, Akira S (2005). Toll-like receptors in innate immunity. Int Immunol.

[16] Lawrence T (2007). Inflammation and cancer: a failure of resolution?. Trends Pharmacol Sci.

[17] Arbour NC, Lorenz E, Schutte BC, Zabner J, Kline JN, Jones M (2000). TLR4 mutations are associated with endotoxin hyporesponsiveness in humans. Nat Genet.

[18] Ferwerda B, McCall MB, Alonso S, Giamarellos-Bourboulis EJ, Mouktaroudi M, Izagirre N (2007). TLR4 polymorphisms, infectious diseases, and evolutionary pressure during migration of modern humans. Proc Natl Acad Sci U S A.

[19] Ferwerda B, McCall MB, Verheijen K, Kullberg BJ, van der Ven AJ, Van der Meer JW (2008). Functional consequences of toll-like receptor 4 polymorphisms. Mol Med.

[20] Hang J, Zhou W, Zhang H, Sun B, Dai H, Su L (2004). TLR4 Asp299Gly and Thr399Ile polymorphisms are very rare in the Chinese population. J Endotoxin Res.

[21] Lin YC, Chang YM, Yu JM, Yen JH, Chang JG, Hu CJ (2005). Toll-like receptor 4 gene C119A but not Asp299Gly polymorphism is associated with ischemic stroke among ethnic Chinese in Taiwan. Atherosclerosis.

[22] Agnese DM, Calvano JE, Hahm SJ, Coyle SM, Corbett SA, Calvano SE (2002). Human toll-like receptor 4 mutations but not CD14 polymorphisms are associated with an increased risk of gram-negative infections. J Infect Dis.

[23] Child NJ, Yang IA, Pulletz MC, de Courcy-Golder K, Andrews AL, Pappachan VJ (2003). Polymorphisms in Toll-like receptor 4 and the systemic inflammatory response syndrome. Biochem Soc Trans.

[24] Faber J, Meyer CU, Gemmer C, Russo A, Finn A, Murdoch C (2006). Human toll-like receptor 4 mutations are associated with susceptibility to invasive meningococcal disease in infancy. Pediatr Infect Dis J.

[25] Su B, Ceponis PJ, Lebel S, Huynh H, Sherman PM (2003). Helicobacter pylori activates Toll-like receptor 4 expression in gastrointestinal epithelial cells. Infect Immun.

[26] Saadat I, Higashi H, Obuse C, Umeda M, Murata-Kamiya N, Saito Y (2007). Helicobacter pylori CagA targets PAR1/MARK kinase to disrupt epithelial cell polarity. Nature.

